# Interprofessional Collaboration and Team Effectiveness of Pharmacists in General Practice: A Cross-National Survey

**DOI:** 10.3390/ijerph20010394

**Published:** 2022-12-26

**Authors:** Thilini Sudeshika, Mark Naunton, Gregory M. Peterson, Louise S. Deeks, Line Guénette, Ravi Sharma, Christopher Freeman, Theo Niyonsenga, Sam Kosari

**Affiliations:** 1 Discipline of Pharmacy, Faculty of Health, University of Canberra, Bruce, ACT 2617, Australia; 2 Department of Pharmacy, Faculty of Allied Health Sciences, University of Peradeniya, Peradeniya 20400, Sri Lanka; 3 School of Pharmacy and Pharmacology, University of Tasmania, Hobart, TAS 7005, Australia; 4 Faculty of Pharmacy, Laval University, Quebec, QC GIV 0A6, Canada; 5 Bedfordshire Hospitals NHS Foundation Trust, Luton LU4 0DZ, UK; 6 School of Pharmacy, Faculty of Health and Behavioural Sciences, University of Queensland, Woolloongabba, QLD 4102, Australia; 7 Faculty of Medicine, The University of Queensland, Herston, QLD 4006, Australia; 8 Metro North Hospital and Health Service, Herston, QLD 4006, Australia; 9 Health Research Institute, Faculty of Health, University of Canberra, Bruce, ACT 2617, Australia

**Keywords:** interprofessional collaboration, pharmacist, team effectiveness, general practice, cross-national survey

## Abstract

As team-based care continues to evolve, pharmacists have been included in general practice teams in many countries, to varying extents, to improve medication use and patient safety. However, evidence on interprofessional collaboration and team effectiveness of pharmacists in general practice is sparse. This study aimed to compare the extent of interprofessional collaboration and team effectiveness of general practice pharmacists in Australia with international sites (Canada and the UK), and identify the factors associated with interprofessional collaboration and team effectiveness. General practice pharmacists from Australia, Canada, and the UK were identified through professional organisations and networks, and invited to participate in an online survey, adapted from existing validated tools. The survey explored interprofessional collaboration through four sub-domains (professional interactions, relationship initiation, trust and role clarity, and commitment to collaboration) and team effectiveness of general practice pharmacists. Of the 101 respondents (26 from Australia, 44 from Canada and 31 from the UK), 79% were female and 78% were aged below 50 years. Interprofessional collaboration and team effectiveness appeared to be high and similar between countries. Total scores for collaboration of pharmacists were 86.1 ± 7.4 in Australia, 88.5 ± 7.5 in the UK, and 89.1 ± 7.3 in Canada (mean ± SD, where higher scores represent more advanced collaboration), while the team effectiveness scores of the pharmacists were 88.6 ± 14.6 in Canada, 91.8 ± 14.6 in Australia and 97.5 ± 14.0 in the UK. Pharmacists who had worked in general practice for a longer time showed advanced interprofessional collaboration while those who worked exclusively in general practice had higher scores for team effectiveness. Overall, general practice pharmacists in the three countries were highly collaborative with general practitioners. Long-term employment and longer work hours could enhance interprofessional collaboration and team effectiveness in general practice pharmacists by improving trust and working relationships over time.

## 1. Introduction

Pharmacists have been included in the general practice/family practice team to support general practitioners (GPs) or family physicians and reduce medication-related risks for patients. Many countries have studied the inclusion of pharmacists in general practice teams and reported that pharmacist-led services in this setting can provide benefits to patients [1,2,3,4,5,6]. The United Kingdom (UK) and Canada are leading countries that have shown a continuous expansion of pharmacists working in general practices [7,8]. In the UK, one key purpose of embedding clinical pharmacists in general practices was to overcome a GP workforce crisis and thereby increase access to health services for patients. Therefore, a two-phase scheme was launched to introduce over 1000 pharmacists to English general practice teams with the support of the National Health Service (NHS) between 2015 and 2019 [9,10]. According to recent reports, this number has now doubled in general practices across the UK [7]. Most pharmacists who work in English general practices have an extended scope of practice, such as prescribing rights [10]. Furthermore, the role description of clinical pharmacists in the UK general practices includes conducting structured medication reviews and improving patient safety and outcomes through a person-centred approach [10]. Studies in the UK have shown that pharmacists can support general practice teams to manage chronic conditions and provide advice for patients who take multiple medicines [9,11,12,13].

Pharmacists have been integrated into Canadian family practices, aiming to improve medication therapy using a collaborative care model. The integration of pharmacists into family practice teams was initiated as a large-scale demonstration project [14,15]. This project was supported by the Ontario Ministry of Health and Long-Term Care through the Primary Health Care Transition Fund between 2004 and 2006 [14]. It is estimated that around 700 pharmacists currently work in family practices across Canada [8]. Provincial and federal government health ministries are supporting the expansion of the co-location of pharmacists in Canadian family practice teams. As a result, most pharmacists in family practice teams are funded by provincial government health authorities [8]. Moreover, Canadian pharmacists have been granted some degree of prescribing rights based on the regulations of the various provinces [16]. Their roles in family practices include performing medication reviews and assessments; developing therapeutic plans with family physicians and patients; and providing education to patients and healthcare professionals [17]. There is an increasing evidence base that shows the advantages of the integration of pharmacists into Canadian family practices [3,18,19,20,21].

The inclusion of pharmacists in Australian general practice teams is a relatively recent development, and the number of pharmacists working in general practices has gradually expanded since 2012 [22]. However, there are still no sustainable funding mechanisms to support the adoption and expansion of pharmacists in general practice teams across Australia [5]. Furthermore, pharmacists in Australia have not been granted prescribing rights, as in Canada and the UK. General practice pharmacists in Australia support GPs and other general practice health professionals to improve the quality use of medicines through collaborative practice. Their roles include performing medication reviews, conducting medicine safety initiatives such as clinical audits, providing education (patients, GPs, and other general practice staff), and liaising with other healthcare providers, but these roles have not been standardised [23]. Even though the expansion of pharmacists working in general practice teams is low across Australia compared to Canada and the UK [24], it has been shown that pharmacists can provide various non-dispensing services to improve patient care [25,26,27].

The value of the collaborative care approach has been recognised in healthcare to improve patient safety. Studies have shown that interprofessional collaboration can help to reduce medication-related risks, optimise medicines, and prevent hospital readmissions [28,29,30]. Furthermore, interprofessional collaboration can help to reduce healthcare costs and inefficiencies [31]. In addition, the collaborative care approach can improve rapport among healthcare professionals and job satisfaction [29,31]. In contrast, a lack of collaboration among healthcare professionals can cause patient dissatisfaction, medication errors, and delayed treatment [32,33]. Thus, collaboration and team effectiveness are key considerations for pharmacists in general practice teams to successfully deliver services to improve patient care.

The literature is sparse with regard to exploring interprofessional collaboration and team effectiveness in general practice following the inclusion of pharmacists. This study was designed to compare the level of interprofessional collaboration and team effectiveness from the viewpoint of pharmacists in general practices in Australia with Canada and the UK, where the number of pharmacists in general practices is much greater. The secondary objective was to identify factors associated with interprofessional collaboration and team effectiveness in the general practice setting.

## 2. Methods

### 2.1. Study Design

**Figure 1:**
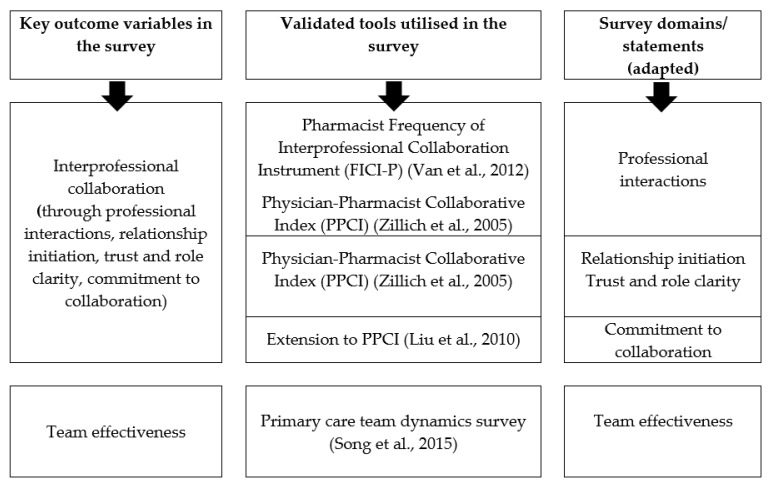
Development of survey instruments [34,35,36,37].

Cross-national survey data were utilised to compare the level of interprofessional collaboration and team effectiveness, and to explore the demographic and contextual factors associated with interprofessional collaboration and team effectiveness, for general practice pharmacists in Australia, Canada, and the UK. The survey questionnaire (File S1) was adapted from previously validated tools, as shown in Figure 1 [34,35,36,37]. The survey instruments explored general practice pharmacists’ (i) professional interactions, (ii) relationship initiation, (iii) trust and role clarity, (iv) commitment to collaboration and (v) team effectiveness. Interprofessional collaboration of pharmacists was assessed through the first four components. An open-ended question was placed in the survey to assess overall comments/suggestions regarding collaboration in general practice. The survey design has been explained in the published protocol [38]. The study was approved by the Human Research Ethics Committee of the University of Canberra (HREC 15-235).

### 2.2. Setting

This survey-based study was conducted in Australia, Canada, and the UK for pharmacists working in general practices from July to December 2020.

### 2.3. Participants, Recruitment, and Data Collection

General practice pharmacists from Australia, Canada, and the UK were invited to participate in an online survey (Qualtrics, Provo, UT, USA). Potential participants were identified through professional networks and contacts in relevant professional organisations already established by the investigators. The survey study was also advertised in newsletters and networks of professional organisations, including the Pharmaceutical Society of Australia, the Primary Care Pharmacy Association (UK), the Royal Pharmaceutical Society of Great Britain, the Primary Care Pharmacy Specialty Network (Canada), and the Quebec Network of Family Medicine Group Pharmacists. The survey was available in English and in French for French-speaking participants from Canada. Survey responses were collected anonymously. When estimating the sample size for a representative sample with a 95% confidence interval and 5% margin of error, 63 responses from Australia (total general practice pharmacists *n* ≈ 75), 323 responses from the UK (total general practice pharmacists *n* ≈ 2000) and 249 from Canada (total family practice pharmacists *n* ≈ 700) were required to adequately reflect the population of general practice pharmacists from each international site [38].

### 2.4. Data Analysis

As the survey instruments were adapted from previously validated tools to assess community pharmacist–GP collaboration, the internal consistency of statements was analysed by using Cronbach’s alpha [39]. The statements of interprofessional collaboration (total items = 22) showed an acceptable internal consistency α = 0.78 and the statements of team effectiveness (total items = 24) showed an excellent internal consistency α = 0.92. Descriptive statistics were used to summarise the demographic details of the participants. Total scores for interprofessional collaboration were calculated through the four sub-domain scores: professional interactions, relationship initiation, trust and role clarity, and commitment to collaboration [34,36]. Overall team effectiveness scores of general practice pharmacists were also calculated for individual participants of the three countries [34,36]. A statistical comparison of survey scores was not performed for the three countries due to the relatively small sample.

As the key outcome variables were respondents’ total scores for interprofessional collaboration and team effectiveness, a multivariable analysis was performed to investigate the association between demographic and other factors (nominal variables) and interprofessional collaboration and team effectiveness of general practice pharmacists [40]. Some categories within factors (age, experience, number of GPs in the general practice, and length of working in the general practice) were merged due to the low number of responses. First, a bivariate analysis was performed to assess the association between the key outcome variables and demographic and other factors. Then, factors which showed an association with the key outcome variables (*p* ≤ 0.05) were included in the final multiple linear regression model.

To assess the relationship between interprofessional collaboration and other factors, all the participants (i.e., all countries) were considered. Length of work in general practice and employment status were the two significant factors from the bivariate analysis included in the final model. In the final model assessing the association between team effectiveness and other factors, only the respondents from Canada and UK were considered (as prescribing rights was a significant factor) and the variables included were country, age, experience, length of working in general practice, employment status and prescribing rights. Regression model was performed for each outcome variable and the unstandardised beta coefficient (B) was reported to reflect the differences in categories with respect to the reference category. Any finding with a *p*-value less than 0.05 was considered as significant. The data analysis was conducted using Statistical Package for the Social Sciences (SPSS ver. 27 IBM Corp, Armonk, New York, NY, USA). Responses to the open-ended question were analysed by using content analysis with the assistance of NVivo qualitative data analysis software (ver. 12, QSR, Melbourne, VIC, Australia) [41,42].

## 3. Results

### 3.1. Demographics

A total of 136 survey responses (29 from Australia, 62 from Canada and 45 from the UK) were received. Of these, 101 responses (26 from Australia, 44 from Canada and 31 from the UK) were included in the analysis after excluding predominantly incomplete responses. Most respondents were female (79%, *n* = 80), and 78% (*n* = 79) were aged below 50 years (Table 1). The majority (55%, *n* = 56) had worked as community pharmacists before working in general practice. Only 36% (n = 36) of pharmacists worked exclusively in general practice (UK *n* = 21, Canada *n* = 11, Australia *n* = 4), while other respondents reported working part-time in multiple settings. Most pharmacists (78%, *n* = 79) had postgraduate qualifications. Among the Australian general practice pharmacists, 58% (*n* = 15) had the qualification of accreditation to perform medication reviews. Most general practice pharmacists 68% (*n* = 51) in both the UK and Canada had prescribing rights. Most respondents from Canada worked in larger general practices (more than 10 GPs in the practice) compared with the respondents from Australia and the UK. Moreover, the majority of pharmacists from Canada and the UK had worked more than 24 months in general practice compared to Australian general practice pharmacists.

### 3.2. Interprofessional Collaboration and Team Effectiveness

Collaboration between the pharmacists and GPs was assessed through four sub-domains including professional interactions, relationship initiation, exchange characteristics (trust and role clarity), and commitment to collaboration (Table 2). Pharmacists’ total scores were, on average, high and similar between countries for both interprofessional collaboration and team effectiveness. Additionally, scores for interprofessional collaboration and team effectiveness were moderately correlated (r = 0.57, *p* < 0.05).

### 3.3. Factors Associated with Collaboration and Team Effectiveness

Only the length of work was a significant factor independently associated with interprofessional collaboration (*p* < 0.05). Collaboration scores were significantly higher in participants who had worked in general practices for more than 24 months than the participants who had worked in general practice for 24 months or less (Table 3). The multiple regression model accounted for 24% of the observed variance (R^2^ = 0.24).

Team effectiveness was significantly associated with the employment status of pharmacists. Participants who worked exclusively in general practices had a higher level of team effectiveness than participants who worked in multiple settings (*p* < 0.05). The multiple regression model accounted for 33% of the observed variance (R^2^ = 0.33).

### 3.4. Comments for the Open-Ended Question

Only 19 (19%) general practice pharmacists (UK = 4, Australia = 5, Canada = 10) completed the open-ended question that provided overall comments/suggestions about collaboration in the survey. Respondents discussed that trust towards the pharmacists and acceptance of pharmacists’ roles by general practice teams could improve the collaboration in general practices/family practices (*n* = 9). Respondents expressed their views on positively influencing collaborative working relationships in general practices through collaboration with nurse practitioners and nurses, communication, and handover between the GP-pharmacist-nurse (*n* = 8). Furthermore, respondents emphasised the requirement of strengthening policies for implementing pharmacists’ roles in the general practice setting (*n* = 2).

## 4. Discussion

This study assessed the extent of collaboration and team effectiveness of general practice pharmacists in Australia compared with general practice pharmacists in Canada and the UK, where this service model is more established and different. The findings indicated that general practice pharmacists in the three countries were highly collaborative with GPs. Furthermore, general practice pharmacists in the three countries rated similarly high scores for relationship initiation with GPs, trust and role clarity, and commitment to collaboration. Our findings suggest that general practice pharmacists were active contributors to initiating collaborative working relationships with GPs. Studies that assessed GP-community pharmacist collaboration has shown similar results for initiating relationships with GPs and establishing trust [43,44]. We observed some demographic and practice factors that were different for general practice pharmacists in the three countries, including the number of GPs in the general practice, employment status and length of employment of pharmacists. This finding probably reflects variations in the organisation of general practices in Australia, Canada, and the UK, and the relative newness of this practice model in Australia.

Our findings indicated that pharmacists who had worked in the general practice for a long time appeared to have an advanced collaboration with GPs, and pharmacists who worked exclusively in general practice reported greater efficiency in general practice teams. Working exclusively in general practice is probably a representative indicator of the pharmacists’ work hours in general practice. Longer work hours and long-term employment may allow more time for pharmacists to improve trust and develop strong working relationships with other general practice team members. Trust has been identified as essential to enhance working relationships, interprofessional collaboration and team performance [44,45,46,47,48,49,50]. In addition, increased work hours in general practice may allow pharmacists to be involved in more multidisciplinary activities to establish their roles in the general practice setting. Increased work hours in general practice could also allow pharmacists to contribute to a greater extent in solving problems, making decisions and thereby, achieving shared goals which could result in improved efficiency in teams [46,51]. Thus, pharmacists who worked exclusively in general practices may be likely to report greater team effectiveness. The findings of this study showed a positive moderate correlation between interprofessional collaboration and team effectiveness. Team effectiveness is an outcome of fruitful interprofessional collaboration [52].

### Strengths and Limitations

This is the first study to assess interprofessional collaboration and team effectiveness in three countries that have implemented general practice pharmacist service models. However, this study has a few limitations to acknowledge. First, we could not reach the pre-specified number of responses to the survey. Thus, our study results may not be generalisable to all general practice pharmacists. In addition, failure to reach the pre-specified number of survey responses may have hindered the analysis of some factors associated with interprofessional collaboration and team effectiveness. There is also potential for non-response bias, and it is not possible to conclude that non-responding general practice pharmacists would have held similar views on collaboration and team effectiveness. Furthermore, pharmacists who responded to this survey might have more advanced interprofessional collaboration than non-respondents. However, this survey was conducted during the COVID-19 pandemic surge when the health workforce had an enormous burden, which may have had an impact on the response rate. Nonetheless, the survey instruments were designed to minimise bias by considering approaches such as utilising Likert scales, ensuring anonymity, creating the survey in two languages (English and French), and adapting statements from a validated tool. Moreover, the data related to interprofessional collaboration were collected only from the pharmacists’ perspective. Thus, further studies are recommended to explore the views of GPs who work with general practice pharmacists.

## 5. Conclusions

General practice pharmacists in Australia, Canada, and the UK were highly collaborative with GPs. Unsurprisingly, long-term employment and longer work hours appear to allow pharmacists to improve trust and develop close working relationships with general practice team members, thereby strengthening interprofessional collaboration and team effectiveness.

## Figures and Tables

**Table 1:** Characteristics of participants.

Characteristic	Australia	Canada	UK
	*n* = 26, (%)	*n* = 44, (%)	*n* = 31, (%)
Age			
20–30 years	2 (8)	9 (20)	5 (16)
31–40 years	8 (31)	15 (34)	11 (35)
41–50 years	11 (42)	10 (23)	8 (26)
51–60 years	2 (8)	8 (18)	5 (16)
More than 60 years	3 (12)	2 (5)	2 (6)
Gender			
Female	21 (81)	32 (73)	27 (87)
Male	5 (19)	12 (27)	4 (13)
Experience as pharmacist			
Less than 5 years	1 (4)	6 (14)	1 (3)
5–11 years	8 (31)	15 (34)	12 (39)
12–18 years	4 (15)	6 (14)	4 (13)
19–25 years	6 (23)	5 (11)	3 (10)
More than 25 years	7 (27)	12 (27)	11 (35)
Background			
Community pharmacy-based practice	14 (54)	24 (55)	18 (58)
Hospital-based pharmacy practice	5 (19)	12 (27)	10 (32)
Other	7 (27)	8 (18)	3 (10)
Accreditation to perform medication reviews	15 (58)	N/A	N/A
Prescribing rights	N/A	27 (61)	24 (77)
Number of GPs in the practice			
1–4	5 (19)	5 (11)	5 (16)
5–10	10 (38)	5 (11)	13 (42)
More than 10	11 (42)	34 (77)	13 (42)
Communication with GPs			
Nil/week	0	0	0
1–2 times/week	7 (27)	14 (32)	5 (16)
3–4 times/week	7 (27)	5 (11)	5 (16)
5 times or more/week	12 (46)	25 (57)	21 (68)
Length of working in general practice			
Less than 6 months	3 (12)	0	1 (3)
6–12 months	4 (15)	3 (7)	4 (13)
13–24 months	8 (31)	5 (11)	3 (10)
More than 24 months	11 (42)	36 (82)	23 (74)
Employment status			
Works only in general practice	4 (15)	11 (25)	21 (68)
Works part-time in multiple settings	22 (85)	33 (75)	10 (32)

Due to rounding, percentages may not always add up to 100%; N/A-Not applicable.

**Table 2:** General practice pharmacists’ scores for the survey.

Domain (Maximum Score)	Australia (*n* = 26) Mean ± SD	Canada (*n* = 44) Mean ± SD	UK (*n* = 31) Mean ± SD
Professional interactions (/20)	12.9 ± 2.3	15.5 ± 3.7	13.1 ± 1.5
Relationship initiation (/15)	13.3 ± 1.6	12.9 ± 2.1	13.1 ± 1.9
Trust and role clarity (/50)	44.0 ± 3.8	44.0 ± 3.8	44.7 ± 3.6
Commitment to collaboration (/20)	15.8 ± 3.1	16.8 ± 2.5	17.4 ± 2.6
Total score for interprofessional collaboration (/105)	86.1 ± 7.4	89.1 ± 7.3	88.5 ± 7.5
Team effectiveness (/120)	91.8 ± 14.6	88.6 ± 14.6	97.5 ± 14.0 ^a^

^a^ In the UK, of 31 participants, only 28 completed the team effectiveness survey.

**Table 3:** Individual factors associated with interprofessional collaboration and team effectiveness of general practice pharmacists.

Demographic Factors	Overall Collaboration			Team Effectiveness ^a^		
	Bivariate Analysis	Multivariable Analysis		Bivariate Analysis	Multivariable Analysis	
	*p*-Value	Beta Coefficient (95% Confidence Interval)	*p*-Value	*p*-Value	Beta Coefficient (95% Confidence Interval)	*p*-Value
Country * Canada	0.27	-	-	**0.05**	−3.90 (−11.04; 3.24)	0.28
Age *	0.86	-	-	**0.03**		
20–30 years					−8.76 (−23.89; 6.37)	0.25
31–40 years					−2.17 (−15.30; 10.97)	0.74
41–50 years					2.13 (−8.08; 12.35)	0.68
Gender (male, female)	0.61	-	-	0.94	-	**-**
Experience *	0.51	-	-	**0.04**		
Less than 12 years					1.78 (−11.12; 14.67)	0.78
12–25 years					−4.24 (−15.38; 6.90)	0.45
Background (community pharmacy, hospital pharmacy, other)	0.58	-	-	0.42	-	-
Number of GPs (less than 10, 10/more than 10)	0.16	-	-	0.06	-	-
Frequency of communication with GPs (1–2 times/week, 3–4 times/week, 5 times or more/week)	0.37	-	-	0.55	-	**-**
Employment status * Only working in general practice	**0.03**	2.56 (−0.17; 5.29)	0.07	**<0.001**	10.11 (2.85; 17.36)	**0.007**
Length of working * More than 24 months	**<0.001**	7.08 (4.26; 9.93)	**<0.001**	**0.01**	6.31 (−2.41; 15.03)	0.15
Accreditation to perform medication reviews ^b^ (Yes, No/not available)	0.06	-	-	0.10	−	**-**
Having rights to prescribe *^,a^ Yes	0.13	-	-	**0.01**	3.79 (−3.08; 10.67)	0.27

* Reference categories: Country (UK), Age (More than 50 years), Experience (More than 25 years), Employment status (Working in other settings), Length of working (24 or less than 24 months), Having rights to prescribe (No/ not available).

^a^ Applicable to general practice pharmacists in UK and Canada: In the final model assessing the association between team effectiveness and other factors, only the respondents from Canada and UK were considered (as prescribing rights was a significant factor).

^b^ Applicable to general practice pharmacists in Australia.

Bold values denote statistical significance at the *p* ≤ 0.05.

## Data Availability

Not applicable.

## Supplementary Materials

The following supporting information can be downloaded at: https://www.mdpi.com/article/10.3390/ijerph20010394/s1, File S1: Collaborative care and team effectiveness survey.
