# Supplementary material for: Interprofessional Collaboration and Team Effectiveness of Pharmacists in General Practice: A Cross-National Survey
Source: Int J Environ Res Public Health. 2022 Dec 26;20(1):394. doi: 10.3390/ijerph20010394 (PMC9819811; doi:10.3390/ijerph20010394)
Supplement: Supplementary file 1 [file ijerph-20-00394-s001.zip › ijerph-2028505-supplementary.pdf]

File S1: Collaborative care and team effectiveness survey for general practice pharmacists

**Collaborative care and team effectiveness survey for pharmacists in general practice/ family Practice (Australia, UK, Canada)**

**Part 1 - Demographic details**

For each question below, write in the answer or select the answer applies to you.

**1. What is your age (Years)?** ☐ 20 – 30 ☐ 31 – 40 ☐ 41 – 50 ☐ 51 – 60 ☐> 60

**2. What is your gender?** ☐ Male ☐ Female ☐ Other ☐ Prefer not to say

**3. How many years of experience do you have as a pharmacist?**

☐ Less than 5 ☐ 5 – 11 ☐ 12 - 18 ☐ 19 – 25 ☐ More than 25

**4. What is your previous working background prior to commencing your career as a practice pharmacist?**

☐ Hospital-based pharmacy practice ☐ Community pharmacy-based practice

☐ Other (please specify) ____________________________________________________________

**5. On average, what proportion (in %) do you currently work in the following areas of pharmacy?**

General practice % ___

Community pharmacy % ___

Hospital pharmacy % ___

Industry % ___

Academia/ research % ___

Total ___

**6. What is your location of your general practice?** (Please write the state/ territory and the suburb in the provided space)- this question is only for the pharmacists in Australia _______________________

**7. What are your academic and professional qualifications?**

_________________________________________________________________________________

**8. Are you annotated as a prescriber? (This question is only included in the surveys for UK and Canada)**

☐ Yes - annotated as an independent prescriber

☐ Yes - annotated as a supplementary prescriber

 Yes - annotated as both an independent and supplementary prescriber

 No

**9. How many general practitioners (GPs) are there in your practice(s)?**

 1 – 4  5 – 9  More than 10

**10. Of the GPs that you are working with, how many do you work with closely? ________________**

**11. How frequently do you contact the GP(s)?**

 Never  1-2 times/week  3-4 times/week  5 times or more/week

**12. What is the most frequent method of communication between you and the GP(s)? (Select all the options that apply for you)**

 Face to face  Telephone  Electronic

 Other (please specify) ____________________________________________________________

**13. How long have you been working in general practice?**

 Less than 6 months  6 - 12 months  12 - 24 months  More than 24 months

**Part 2 - Professional interactions**

For each statement below, select the answer that applies to you.

**14.** How frequently did the following activities occur in the **LAST MONTH?**

**I contacted a GP to discuss a patient's medicine-related problem(s)**

 Nil  1 - 2 times  To some extent  3-4 times  5 times or more

**I informed a GP of new products/services available**

 Nil  1 - 2 times  To some extent  3-4 times  5 times or more

**I was contacted by a GP for medicine information**

 Nil  1 - 2 times  To some extent  3-4 times  5 times or more

**I was contacted by a GP to discuss a patient's medicine-related problem(s)**

 Nil  1 - 2 times  To some extent  3-4 times  5 times or more

**I received a referral from a GP (to review medicines/ educate/ assess adverse effects/provide more information of medicines)**

 Nil  1 - 2 times  To some extent  3-4 times  5 times or more

**15.** How frequently did you receive referrals from the following personnel in the **LAST MONTH**?

**Patients**

□ Nil □ 1 - 2 times □ To some extent □ 3-4 times □ 5 times or more

**Nurse**

□ Nil □ 1 - 2 times □ To some extent □ 3-4 times □ 5 times or more

**Psychologist/ mental health service**

□ Nil □ 1 - 2 times □ To some extent □ 3-4 times □ 5 times or more

**Dietitian**

□ Nil □ 1 - 2 times □ To some extent □ 3-4 times □ 5 times or more

**Reception staff**

□ Nil □ 1 - 2 times □ To some extent □ 3-4 times □ 5 times or more

**Care home manager/ support staff**

□ Nil □ 1 - 2 times □ To some extent □ 3-4 times □ 5 times or more

**Community pharmacist**

□ Nil □ 1 - 2 times □ To some extent □ 3-4 times □ 5 times or more

**Hospital pharmacist**

□ Nil □ 1 - 2 times □ To some extent □ 3-4 times □ 5 times or more

**Other allied health professional e.g., physiotherapist, optometrist**

□ Nil □ 1 - 2 times □ To some extent □ 3-4 times □ 5 times or more

**Community organisations/ providers**

□ Nil □ 1 - 2 times □ To some extent □ 3-4 times □ 5 times or more

**Part 3 - Exchange characteristics (Relationship initiation, role specification, trustworthiness)**

Complete this part of the survey based on your overall experience of working together with GP(s) to improve patient care.

**16.** Please select the answer that applies to you. (N/A- Not Applicable)

**I spent time trying to learn how I can help a GP to provide better care**

□ Not at all □ To a less extent □ To some extent □ To moderate extent □ To a great extent □ N/A

**I provided information to a GP about a specific patient**

□ Not at all □ To a less extent □ To some extent □ To moderate extent □ To a great extent □ N/A

**I showed an interest in helping a GP to improve his/her practice**

□ Not at all □ To a less extent □ To some extent □ To moderate extent □ To a great extent □ N/A

**17.** Please select the answer that applies to you. (N/A- Not Applicable)

**A GP is a credible practitioner**

□ Strongly disagree □ Disagree □ Neutral □ Agree □ Strongly agree □ N/A

**My interactions with a GP are characterised by open communication of both parties**

□ Strongly disagree □ Disagree □ Neutral □ Agree □ Strongly agree □ N/A

**I count on GP(s) to do what he/she says**

□ Strongly disagree □ Disagree □ Neutral □ Agree □ Strongly agree □ N/A

**I intend to keep working together with GP(s)**

□ Strongly disagree □ Disagree □ Neutral □ Agree □ Strongly agree □ N/A

**I trust the GP's drug expertise**

□ Strongly disagree □ Disagree □ Neutral □ Agree □ Strongly agree □ N/A

**Communication between a GP and me should be two-way**

□ Strongly disagree □ Disagree □ Neutral □ Agree □ Strongly agree □ N/A

**In providing patient care, I need GP(s) as much as GP(s) need(s) me**

□ Strongly disagree □ Disagree □ Neutral □ Agree □ Strongly agree □ N/A

**I should work with GP(s) to overcome disagreements on my role in managing medicines**

□ Strongly disagree □ Disagree □ Neutral □ Agree □ Strongly agree □ N/A

**A GP and I should be mutually dependent on each other in caring for patients**

 Strongly disagree  Disagree  Neutral  Agree  Strongly agree  N/A

**A GP and I should negotiate to come to an agreement on my activities in managing medicines**

 Strongly disagree  Disagree  Neutral  Agree  Strongly agree  N/A

**Part 4 – Commitment to collaboration**

Complete this part of the survey based on your overall experience of working together with GP(s) to improve patient care.

**18.** Please select the answer that applies to you. (N/A- Not Applicable)

**There is cooperation between a GP and pharmacist in managing medicines of our patients**

 Strongly disagree  Disagree  Neutral  Agree  Strongly agree  N/A

**Both GP's and pharmacist's options are considered in making decisions for our patients**

 Strongly disagree  Disagree  Neutral  Agree  Strongly agree  N/A

**Decision making is coordinated between a GP and pharmacist**

 Strongly disagree  Disagree  Neutral  Agree  Strongly agree  N/A

**Decision making responsibilities for the patient's medicines are shared between a GP and pharmacist, whenever necessary**

 Strongly disagree  Disagree  Neutral  Agree  Strongly agree  N/A

**19.** If you have any other comments or suggestions regarding collaborative care, please write in the provided space.

____________________________________________________________

____________________________________________________________

____________________________________________________________

**PART 5 - Team effectiveness** (These statements were combined with collaborative care survey at the time of distribution).

Please select the answer that applies to you. (N/A- Not Applicable)

**1. Membership of my team changes so frequently that we don't really have a team**

☐ Strongly disagree ☐ Disagree ☐ Neutral ☐ Agree ☐ Strongly agree ☐ N/A

**2. My team has the right "mix" of members—a group of people who bring different clinical perspectives and experiences to the work**

☐ Strongly disagree ☐ Disagree ☐ Neutral ☐ Agree ☐ Strongly agree ☐ N/A

**3. It is clear to my team what behaviour is acceptable / not acceptable**

☐ Strongly disagree ☐ Disagree ☐ Neutral ☐ Agree ☐ Strongly agree ☐ N/A

**4. Our practice recognises and reinforces teams that perform well**

☐ Strongly disagree ☐ Disagree ☐ Neutral ☐ Agree ☐ Strongly agree ☐ N/A

**5. My team has goals that are clear, useful, and appropriate to my practice**

☐ Strongly disagree ☐ Disagree ☐ Neutral ☐ Agree ☐ Strongly agree ☐ N/A

**6. There is a desire among team members to work collaboratively**

☐ Strongly disagree ☐ Disagree ☐ Neutral ☐ Agree ☐ Strongly agree ☐ N/A

**7. If asked, I could explain every team member's role and how they overlap**

☐ Strongly disagree ☐ Disagree ☐ Neutral ☐ Agree ☐ Strongly agree ☐ N/A

**8. My team encourages patients to be active participants in decisions about their care**

☐ Strongly disagree ☐ Disagree ☐ Neutral ☐ Agree ☐ Strongly agree ☐ N/A

**9. My team does a good job of helping patients understand their care plan**

☐ Strongly disagree ☐ Disagree ☐ Neutral ☐ Agree ☐ Strongly agree ☐ N/A

**10. The patient's needs and preferences are treated as an essential part of my team's decisions**

☐ Strongly disagree ☐ Disagree ☐ Neutral ☐ Agree ☐ Strongly agree ☐ N/A

**11. Each team member shares accountability for team decisions and outcomes**

☐ Strongly disagree ☐ Disagree ☐ Neutral ☐ Agree ☐ Strongly agree ☐ N/A

**12. My team has developed effective strategies for sharing patient treatment goals among team members**

☐ Strongly disagree ☐ Disagree ☐ Neutral ☐ Agree ☐ Strongly agree ☐ N/A

**13. Relevant information about changes in patient status or care plan is reported to the appropriate team member in a timely manner**

 Strongly disagree  Disagree  Neutral  Agree  Strongly agree  N/A

**14. All team members effectively use the patient health record as a communication tool**

 Strongly disagree  Disagree  Neutral  Agree  Strongly agree  N/A

**15. My team addresses patients' concerns effectively through team meetings and discussions**

 Strongly disagree  Disagree  Neutral  Agree  Strongly agree  N/A

**16. Team meetings provide an open, comfortable, safe place to discuss concerns**

 Strongly disagree  Disagree  Neutral  Agree  Strongly agree  N/A

**17. My team has an effective process for conflict management**

 Strongly disagree  Disagree  Neutral  Agree  Strongly agree  N/A

**18. Overall, members of our team do a very good job of coordinating their different patient-related jobs and activities**

 Strongly disagree  Disagree  Neutral  Agree  Strongly agree  N/A

**19. Members of my team act upon the information I communicate to them**

 Strongly disagree  Disagree  Neutral  Agree  Strongly agree  N/A

**20. The way my team members interact makes the delivery of care highly efficient**

 Strongly disagree  Disagree  Neutral  Agree  Strongly agree  N/A

**21. The way my team members interact is very good for the quality of patient care**

 Strongly disagree  Disagree  Neutral  Agree  Strongly agree  N/A

**22. Working on a team like mine keeps members of my team enthusiastic and interested in their jobs**

 Strongly disagree  Disagree  Neutral  Agree  Strongly agree  N/A

**23. I feel integral to my team**

 Strongly disagree  Disagree  Neutral  Agree  Strongly agree  N/A

**24. I experience excellent teamwork with the members of my team**

 Strongly disagree  Disagree  Neutral  Agree  Strongly agree  N/A

***Thank you for your time and interest in completing the survey.***
